# Lifestyle impact on migraine during home confinement

**DOI:** 10.1007/s13760-021-01856-2

**Published:** 2022-02-11

**Authors:** Antonio Granato, Giovanni Furlanis, Laura D’Acunto, Sasha Olivo, Alex Buoite Stella, Paolo Manganotti

**Affiliations:** Clinical Unit of Neurology, Department of Medicine, Surgery and Health Sciences, Headache Centre, University Hospital and Health Services of Trieste - ASUGI, University of Trieste, Strada Di Fiume, 447, 34149 Trieste, Italy

**Keywords:** COVID-19, Lockdown, Headache, Migraine, Lifestyle, Home confinement

## Abstract

**Background:**

The COVID-19 lockdown has influenced people lifestyle, behaviour, physical activity (PA), and working habits as well as, possibly, migraine. The aim of the study was to assess the impact of lockdown on the burden of migraine attacks during COVID-19 lockdown.

**Methods:**

Patients were interviewed, and data about demographics, PA, daily behaviour, working habits, disability (HIT-6) and characteristics of migraine and drugs consumption were compared between the first month of the lockdown in Italy (March 2020), and a reference month prior the lockdown (January 2020).

**Results:**

37 patients were analysed, classified as migraine without aura (MwoA) (*n* = 26) and migraine with aura (MwA) *plus* migraine with and without aura (MwA/MwoA) (*n* = 11). During the lockdown, a greater proportion of patients with insufficient PA (65% vs 31%; *p* = 0.012) were found. Reduced mean headache duration [3 h, (2–12) vs 2 h (1–8); *p* = 0.041] and HIT score [59 (51–63) vs 50 (44–57); *p* = 0.001] were found in MwoA patients during the lockdown, while no changes found in patients with MwA + MwA/MwoA.

**Conclusions:**

Lockdown induced significant changes in PA and working habits of people with migraine and was found to be associated with improved migraine-related symptoms which might depend by different lifestyle habits.

## Introduction

In December 2019 several cases of interstitial pneumonia caused by a novel coronavirus (SARS-CoV2) have been identified in Wuhan, Hubei region, China. The WHO declared Coronavirus Disease 19 (COVID-19) pandemic on March 11th following the rapid spread of the infection to Europe and America. The most involved countries in the outbreak imposed strict mobility restrictions to limit unnecessary contacts between people and consequently the diffusion of the COVID19. Italy was one of the earliest countries to record a COVID19 case in Europe. It recorded its first case on 20th February 2019 in Codogno, Lombardy [1]. On March 9th, after a fast diffusion of the infection with a total of 9172 confirmed cases, Italy announced a lockdown measure that lasted until May 3rd (data from Italian Health Ministry daily official report, see http://www.salute.gov.it/portale/home.html). During this period, leaving home was granted only to those with essential jobs, to buy necessities or to access medical care for urgent health issues. Those restrictions decreased the mobility of the Italian population and profoundly affected daily habits, as well as sleep duration and quality, food intake and physical activity [2]. Many factors can trigger migraine attacks, as well as the menstrual cycle, weather changes, sleep disturbances, eating habits, stress and physical activity [3, 4]. Kelman et al. showed significant differences between trigger factors in patients with Migraine with Aura (MwA) and Migraine without aura (MwoA) [5]. Lockdown represented a significant disruption to the daily routines and lived experiences of Italian people. Lockdown and the relevant lifestyle changes could affect the frequency of the headache attacks of migrainous patients [6].

The aim of the study was to assess the impact of lockdown in the burden of migraine attacks during COVID-19 lockdown. We investigated some of the biological and behavioural changes occurring in daily life and health (as physical activity, diet, work and sleep) during the COVID-19 lockdown and their relationship to migraine patterns.

## Materials and methods

### Participants and protocol

Data were collected using a phone interview model developed by the authors. The Phone interviews were carried out according to the standardized emergency telemedicine protocol planned by the Headache Centre of ASUGI Trieste. Inclusion criteria were patients evaluated in the Headache Centre of ASUGI Trieste from 01.06.2019 to 31.12.2019 and diagnosed as episodic migraine with or without aura, age > 18 y, residence in Friuli-Venezia Giulia region during the COVID-19 lockdown. At the time of the interview, all the included patients were not assuming prophylactic therapy or they were on prophylaxis with the same therapy from at least 3 months. Exclusion criteria were spontaneous modifications of the prophylactic therapy, including starting a new therapy, disabling comorbidities, diseases that caused walking impairment, moderate/severe cognitive impairment or psychiatric conditions, and current or past positivity to COVID-19. Sample size was based on the number of patients with the specific clinical features described above living in the same province and who participated in the interview during the limited early first lockdown period to obtain homogenous results. The interview was performed by neurologists specialized in headache. The analysis of migraine-related items and the statistical analysis were conducted by a co-author with experience questionnaires analysis and statistics.

## Survey design

To the best of the authors' knowledge, no previously validated questionnaire was available to assess the variety of information related to behaviours and health during lockdown in migrainous patients. Therefore, a survey was designed by a multidisciplinary team including a physiologist and a neurologist expert in headache, and it was based on previous experience with online surveys [7]. A pilot testing was conducted on five patients (not included in this study) on two separate occasions 10 days apart. The reliability of the survey was investigated by means of test–retest correlation that confirmed the appropriate reliability (Pearson’s or Spearman’s *r* from 0.567 to 1.000, *p* < 0.05) (unpublished data). After confirming the willingness to participate and the acceptance of the informed consent and data protection, participants were asked some demographic data including gender (with the options of non-binary and not responding), age, height, body mass index (BMI), profession (student, employee, manager, freelance, unemployed, housewife, retired/other, etc.). Responders were also asked whether they continue to work as usual during the lockdown, or whether they stayed at home and could not work due to Italian ministerial restrictions, not even in remote working. Questions about the habitation included mean size, if it was a house or an apartment, if the habitation had a garden or a roof, and if it was set in an urban or rural area. To investigate previous physical activity (PA) practices, a question regarded whether prior to the lockdown they were involved in any structured PA program such as joining a gym, swimming pool or sport club at least 2 times/week and for longer than 3 months, or not (n-PA), and how many training/week they performed. Participants were also asked to rate if during the lockdown period they ate more, the same, or less than usual.

The second section of the interview included questions referring to a reference month prior the lockdown—January (Reference). Participants were asked to check on their smart technology device the mean daily step count of the reference month. Mean daily sleep hours over the reference month could be reported both as results of a smart technology device, or by personal recall. Sleep quality could be rated as poor, fair, nor bad nor good, good, or excellent. Participants were asked to recall and report their fluid and beverages intake, namely: (i) daily fluid intake (mL), (ii) daily units of coffee, (iii) alcohol consumption and daily units of alcohol (wine/spirits). Smoking habits were investigated, asking the daily smoked cigarettes. To assess PA in terms of metabolic equivalents (METs), a validated version of the short form of the International Physical Activity Questionnaire (IPAQ-SF) was used. [8] Participants were divided into an “insufficient” or “sufficient” daily PA group based on the cutoff of 10,000 steps [9] or 700 MET [8]. Working habits were investigated asking if they were in remote working modality, how many hours/day they worked (at the job place or at home), and how many time they spent in front of a screen to work (at the job place or at home).

At last, questions about migraine-related health outcomes were investigated. Patients were asked to report (i) their prophylactic therapy during the period of reference, (ii) the number of migraine episodes in a month (severe vs mild episodes) and mean episode duration, (iii) what type of symptomatic therapy they used, how many times in a month, and its efficacy (complete or not), (iv) the items to compute the Headache Impact Test (HIT-6) score.

The third and last part of the interview asked the same questions of the second section yet referring to the first month of lockdown in Italy between 9th of March and 9th of April 2020.

## Statistical analysis

All statistical analyses were performed using SPSS Statistics 23 (IBM, Armonk/NY, USA). Descriptive statistics included medians and interquartile range (IQR) for continuous variables and proportions for categorical variables (n, %). Differences between the outcomes during the reference month and during the lockdown month were evaluated with the Wilcoxon Signed Rank Test for continuous variables and the McNemar test for categorical variables. Since of the similar annual aura episodes frequency, MwA and MwA/MwoA were grouped in the same category for statistical analysis. Subgroup comparisons were performed between MwoA and MwA + MwA/MwoA, and between groups based on the working condition during the lockdown period, living in an urban/rural area, improved/worsened sleep quality during the lockdown, and living in a house/apartment.

Between-group comparisons were performed with the two-tailed Mann–Whitney *U* Test for continuous variables and chi-square test for categorical variables. A significance value was selected for *p* < 0.05. Bonferroni correction for multiple testing was applied and adjusted p values presented accordingly.

## Results

During the recruitment period, 56 individuals were selected, and 44 responded to the phone interview. Among them, 7 were excluded for incomplete responses or spontaneous start of a new prophylactic therapy. Thirty-seven were included in the final analysis. The participants' demographic characteristics are presented in Table 1 for MwoA (*n* = 26) vs MwA + MwA/MwoA (*n* = 11). No significant differences between groups were present for the demographic variables.

**Table 1:** Participants’ demographics. Data are presented as medians (interquartile range) and frequencies

Personal characteristics	MwoA *n* = 26	MwA + MwA/MwoA *n *= 11
Age [y]	45 (31–53)	38 (26–47)
Females [*n* (%)]	23 (88)	7 (64)
BMI [kg/m^2^]	24 (18–32)	27 (18–50)
Habitation [*n* (%)]		
Urban area	20 (77)	9 (82)
Town/rural area	6 (23)	2 (18)
House	9 (35)	4 (36)
Apartment	17 (65)	7 (64)
Garden/Roof	17 (65)	5 (45)
Size of the house [m^2^]	94 (40–250)	85 (60–150)
≥ 90 m^2^ [*n* (%)]	13 (50)	4 (36)
< 90 m^2^ [*n* (%)]	13 (50)	7 (64)
Occupation [*n* (%)]		
Occupied	19 (73)	11 (100)
Manager	2 (8)	1 (9)
Employee	10 (38)	5 (45)
Worker	2 (8)	2 (19)
Freelance	5 (19)	3(27)
Non occupied/Retired/Student	7 (27)	0 (0)
Occupation during lockdown		
Unchanged	7 (27)	2 (19)
Suspended	6 (23)	4 (36)
Smart-working	6 (23)	5 (45)
Training [days/week]	0 (0–1)	0 (0–2)
Structured PA [*n* (%)]	6 (23)	4 (36)
Food habits lockdown [*n* (%)]		
More than usual	9 (35)	5 (45)
Same as usual	12 (46)	6 (55)
Less than usual	5 (19)	0

*MwoA* migraine without aura; *MwA* migraine with aura; *BMI* Body Mass Index; *PA* Physical Activity

## Physical activity, behavioural and working habits modifications during the lockdown period

In the MwoA group, mean daily step count decreased from 10,000 (7000–12,000) steps during the reference period, to 2000 (1000–2000) steps during the lockdown (*p* = 0.018; with Bonferroni correction *p* = 0.036). Physical activity changed, with a reduction of daily METs (792, 531–1950 vs 273, 82–816 MET) (*p* < 0.001 with Bonferroni correction *p* < 0.001), which led to a higher proportion (65% vs 31%) of patients with insufficient physical activity levels (*p* = 0.012 with Bonferroni correction *p* = 0.024). Sleep duration and sleep quality were not affected by the lockdown period, as well as fluid intake, alcohol consumption, or smoking habits. Only coffee consumption was found different, by a tendency to reduce the units of daily coffee consumption during the lockdown (2, 1–3 vs 1, 1–2) (*p* = 0.048; with Bonferroni correction *p* = 0.096). Working habits changed during the lockdown, with a significant reduction of the time spent working outside the habitation (*p* = 0.001; with Bonferroni correction *p* = 0.002) that was compensated by an increase of remote working (*p* = 0.011, with Bonferroni correction *p* = 0.022).

In the MwA + MwA/MwoA group, no significant difference was present for sleep characteristics, nor fluid intake, alcohol consumption, smoking habits, as well as coffee consumption. Steps count and daily METs decreased during the lockdown, although without reaching statistical significance (*p* = 0.068 and *p* = 0.074, respectively; with Bonferroni correction *p* = 0.136 and *p* = 0.148). Working habits changed during the lockdown, with a significant reduction of the time spent working outside the habitation (*p* = 0.005; with Bonferroni correction *p* = 0.010) that was partially compensated by an increase of remote working (*p* = 0.039; with Bonferroni correction *p* = 0.78). Comparisons of the variables in the MwoA and MwA + MwA/MwoA groups between the reference and the lockdown period are shown in Table 2.

**Table 2:** Participants’ characteristics during the reference month (January) and during the lockdown (April). Data are presented as medians [interquartile range] and frequencies

Personal Characteristics	MwoA *n* = 26			MwA + MwA/MwoA *n* = 11		
	Reference	Lockdown	Sig	Reference	Lockdown	Sig
Sleep						
Duration [h]	7 (6–8)	7 (6–8)	0.267	6 (6–8)	7 (6–8)	0.450
Quality [*n* (%)]			0.453			1.000
Poor	0	0		0	0	
Fair	5 (19)	8 (31)		2 (18)	1 (10)	
Not bad nor good	10 (38)	5 (19)		4 (36)	1 (10)	
Good	10 (39)	11 (42)		4 (36)	5 (45)	
Excellent	1 (4)	2 (8)		1 (10)	4 (35)	
Daily Fluid Intake [mL]	1500 (1000–2000)	1500 (1100–2000)	0.590	1500 (1125–2250)	1500 (1125–1875)	0.892
Coffee [*n*]	2 (1–3)	1 (1–2)	0.048	3.5 (2–5)	3.5 (2–5)	1.000
Smokers [*n* (%)]	8 (31)	7 (27)	1.000	4 (36)	4 (36)	1.000
Cigarettes [*n*/day]	5 (3–10)	5 (3–8)	0.317	8 (4–18)	12 (4–20)	0.317
Usual alcohol consumers [*n* (%)]	15 (58)	12 (46)	0.375	9 (82)	7 (73)	1.000
Wine/beer [U/week]	1 (0–1)	0 (0–1)	0.084	1 (0–1)	1 (0–1)	0.705
Spirits [U/week]	0 (0–0)	0 (0–0)	0.317	0 (0–0)	0 (0–0)	0.317
Work						
Work at the workplace [h/day]	8 (5–8)	0 (0–6)	**0.001**	6 (5–8)	0 (0–0)	**0.005**
Screen use at the workplace [h/day]	1 (0–6)	0 (0–0)	**0.011**	3 (0–8)	0 (0–0)	**0.011**
Remote working at home [h/day]	0 (0–0)	0 (0–6)	**0.011**	0 (0–0)	0 (0–8)	0.039
Screen use remote working at home [h/day]	0 (0–0)	0 (0–6)	**0.011**	0 (0–0)	0 (0–8)	0.039
Overall screen use [h/day]	0 (0–6)	0 (0–6)	1.000	3 (0–8)	0 (0–8)	0.465
Daily Steps Count [count]	10,000 (7000–12,000)	2000 (1000–2000)	**0.018**	8000 (5250–13,750)	2000 (1250–2000)	0.068
IPAQ-SF (MET)	792 (531–1950)	273 (82–816)	** < 0.001**	1356 (495–2142)	426 (66–2209)	0.074
Insufficient MET levels [*n* (%)]	8 (31)	17 (65)	**0.012**	5 (45)	7 (64)	0.625
Headache						
Total headache days [count]	4 (3–8)	3 (1–6)	0.521	2 (1–4)	2 (1–5)	0.757
Severe headache days	2 (1–3)	1 (0–3)	0.376	1 (0–2)	1 (0–2)	0.762
Duration of attack (h)	3 (2–12)	2 (1–8)	0.041	2 (1–2)	2 (1–2)	0.893
Symptomatic drugs	3 (1–4)	2 (1–4)	0.812	2 (0–3)	1 (1–5)	0.676
HIT-6	59 (51–63)	50 (44–57)	**0.001**	56 (49–60)	45 (36–49)	0.062
Pain free after SDI [*n* (%)]	13 (50)	20 (77)	0.070	3 (27)	5 (45)	1.000

*MwoA* migraine without aura; *MwA* migraine with aura; *IPAQ*-*SF* International Physical Activity Questionnaire-Short Form; *MET* metabolic equivalents; *HIT* Headache Impact Test. *SDI* symptomatic drug intake. Significance value (Sig.) for reference-lockdown comparison. Bold values for comparisons remaining significant after Bonferroni correction for multiple testing

## Headache characteristics modifications during the lockdown period

In the MwoA group, during the lockdown a reduction in mean headache episode duration was reported (3, 2–12 vs 2, 1–8 h) (*p* = 0.041; with Bonferroni correction *p* = 0.082), as well as a significant decrease in the HIT score (59, 51–63 vs 50, 44–57) (*p* = 0.001; with Bonferroni correction *p* = 0.002). In contrast, no differences were reported in the MwA + MwA/MwoA group, suggesting no effects of the lockdown in this population. Comparisons in headache characteristics between the reference period and the lockdown are shown in Table 2.

## Subgroup analyses

When living area was compared (Urban or Rural), only in the MwoA group a tendency for a higher HIT-6 score during the lockdown was reported for the patients living in an urban area compared to those living in a rural area (53, 46–57 vs 42, 36–49) (*p* = 0.033; with Bonferroni correction *p* = 0.066). Living in a house compared to living in an apartment influenced MwoA headache characteristics prior to the lockdown but not during the lockdown. Indeed, MwoA who were living in a house showed a tendency for a less symptomatic drugs consumption compared to MwA + MwA/MwoA patients living in an apartment (1, 1–3 vs 3, 2–4) (*p* = 0.026; with Bonferroni correction *p* = 0.52). Effects of improved sleep quality (i.e., those who improved by at least one point their sleep quality self-assessment) suggested no influence of any of the outcomes in both groups.

## Discussion

During the lockdown, we observed a significant reduction in duration of migraine attack and the significant reduction of symptomatic drugs in patients with migraine. These results are in line with other findings collected in larger samples suggesting the course of headache had similar frequency but decreased severity and duration [10] and in general improved migraine features during the pandemic [6, 11, 12]. During the state of emergency, the attention of healthcare providers and health authorities was primarily focused on infected patients and the frontline responders. This also had an impact on other services dedicated to disabling pathologies, such as migraine. In fact, lockdown’s sanitary restrictions have reduced the rate of traditional neurologist visits for non-acute headache and conversely strengthened the telemedicine consultations, to help migraine patients and overall to maximize social distancing [13]. COVID 19 pandemic is a challenge for each and every one of us, in particular for those with a historical lower tolerance threshold for stressful events, as it seems to be the case with migraineurs [14]. Even if response to stress was thoroughly researched [15], this epochal event offers the possibility to study for the first time the adaptation of migraine patients to an unprecedented calamity of unknown duration and its direct effects on lifestyle habits and headache.

In this study, we investigated the effects of lifestyle change during lockdown period on patients of our Headache Centre suffering with episodic migraine (both MwoA and mixed MwA/MwoA). The sample was determined to assess the biological and behavioural changes occurring in daily life and health during the COVID-19 lockdown and their relationship to migraine patterns. Through a series of telephone interviews conducted using a structured questionnaire, run in full lockdown’s phase one (in April), we tested the chances occurred in course of lockdown period in terms of daily behavioural patterns, work activity and migraine characteristics, such as frequencies of attacks, pain intensity, drugs assumption and disability (HIT-6 score).

Our results evidenced that MwoA group showed a tendency to amelioration in attack duration and a significant improvement of the HIT-6 score compared to pre-lockdown period, while no positive or negative effects of lockdown were observed in MwA + MwA/MwoA patients. The attack duration decreased during lockdown, probably because of the increased time spent at home with less stressors and with the possibility to rest during the crisis. The HIT-6 questionnaire is a validate screening instrument to assess headache severity and changes in a patient’s clinical status over a short period of time [16], taking individual patients’ level as well as variability in different populations into account. The HIT-6 score reduction in MwoA patients revealed a reduction of the impact of headache on patient’s life despite this dramatic scenario. Subgroup analysis revealed a lower HIT score in MwoA patients living in rural areas compared to those living in urban areas. Moreover, MwoA patients living in houses used less symptomatic drugs compared to MwA + MwA/MwoA patients living in apartments. This data suggests a possible influence of living area on migraine, especially when home confinement is necessary. Living in a rural area and in a house and not in an apartment seem to be protective factors for patients suffering from migraine without aura, while migraine with aura seems to be less affected by the home location. To the best of the authors' knowledge, this is the first study investigating the effect of housing conditions on the occurrence of migraine disorders in times of pandemics. Total headache days included also severe ones, and drugs assumption did not change in both MwoA and MwA + MwA/MwoA groups.

Food and fluid intake, smoke and alcohol did not significantly change during lockdown, probably due to the habit of migraine patients to avoid well-established headache triggers [5]. The data confirm that regular lifestyle behaviour is associated with no increasing of migraine attacks, as previously demonstrated [17]. Home confinement did not result in sleep disruption in our population, and no significant modifications of sleep quality and duration were observed. Sleep disruption is often linked to exacerbation of headache [18–20]. In our sample, the absence of sleep disturbance may partially justify the stability of migraine patterns.

As expected, a reduction of PA was detected in MwoA patients during lockdown, in line with data collected in a large sample of Italian adults, which showed a good agreement with data obtained from smart technology devices [21]. The data from the Nord-Trøndelag Health Surveys (HUNT) suggests that physical inactivity increased the risk of headache and, more interestingly, that hard physical exercise 1–2 h per week reduced the risk of migraine compared to inactivity [18, 19]. PA was also associated to headache disorders independent of economic and psychosocial factors [20]. A recent review found that aerobic exercise reduces the number of migraine days and pain intensity [21]. However, in our patients, we did not observe a clear dose–response relation between reduction of PA and headache. Conversely, no modification of physical activity was found among our 11 MwA + MwA/MwoA patients. Due to the cross-sectional nature of the study, it is not possible to determine if physical activity influenced migraine characteristics or if migraine features had an attack on physical activity. As suggested by *Hagen *et al., we could speculate on the fact that “*individuals who had already experienced severe headache or migraine attacks may avoid exercise (reversed causality)*” [18].

In both groups of patients, working habits changed during the lockdown, with a significant reduction of the time spent working outside the habitation that was compensated by an increase of remote working. This shift also concerns video display terminal use. Ten patients reported a complete suspension of their job activity. The effect of work on the occurrence of headache disorders is always difficult to assess. A cross sectional study of Swedish population found that dissatisfaction with work and worries about losing one’s job were conditions that had a strong association with headache disorders [20]. In an Italian study, severe workplace problems were associated with higher disability level in migraineurs; however, no associations between higher MIDAS and higher work-related difficulties were demonstrated in episodic migraineurs [22]. Finally, an interesting longitudinal Brazilian study has shown that among women definite migraine was associated with time and strain-based interference of work with family, interference of family with work and lack of time for personal care and leisure [23]. A balance between working activity and domestic demand is not always simple to maintain, and this can have a negative impact on migraine disorders, especially among women. However, during lockdown, work activity did not have negative effect on our population. This fact could be explained by the reduction of job-strain interference on life, more time availability also for self-care and the possibility of home resting in case of headache attack [11, 12].

Although stressful events have demonstrated association with migraine attack onset among people with episodic migraine [15], restrictions and lifestyle changing due to COVID 19 pandemic does not seem to have affected our migraine population. A possible explanation could be that Trieste, mainly the rural area, was one of Northern provinces of Italy with fewer incidence of COVID 19 cases, so the impact on population was less dramatic. From a psychological point of view, the reduction of disability due to migraine could represent a coping strategy for minimising stress in difficult situations [24].

The principal limitations of this survey are the retrospective design, the small sample of population (although well selected and representative of an area with similar lifestyle and pandemic-related characteristics), and the lack of specific investigation of psychological sphere related to job-stress and perceived stress during lockdown.

To summarize, attack duration and disability of migraine significantly improved in the group of MwoA patients during lockdown. Habitat comfort such as living in a house and in rural areas might have a protective role. Daily behavioural patterns, including food assumption, hydration status, alcohol, smoke, and sleep, did not significantly change during the home confinement in both groups and they have not been associated to any modification of migraine patterns. During the lockdown, patients reduced PA and modified working habits without impact on migraine characteristics.

Home confinement due to COVID lockdown offered the opportunity to investigate for the first time the adaptation of migraine to an unprecedented calamity, which directly modified behaviour, lifestyle, interpersonal relationships and working activity.
